# Particle Size Controls on Water Adsorption and Condensation Regimes at Mineral Surfaces

**DOI:** 10.1038/srep32136

**Published:** 2016-08-26

**Authors:** Merve Yeşilbaş, Jean-François Boily

**Affiliations:** 1 Department of Chemistry, Umeå University, SE-901 87 Umeå, Sweden

## Abstract

Atmospheric water vapour interacting with hydrophilic mineral surfaces can produce water films of various thicknesses and structures. In this work we show that mineral particle size controls water loadings achieved by water vapour deposition on 21 contrasting mineral samples exposed to atmospheres of up to ~16 Torr water (70% relative humidity at 25 °C). Submicrometer-sized particles hosted up to ~5 monolayers of water, while micrometer-sized particles up to several thousand monolayers. All films exhibited vibrational spectroscopic signals akin to liquid water, yet with a disrupted network of hydrogen bonds. Water adsorption isotherms were predicted using models (1- or 2- term Freundlich and Do-Do models) describing an *adsorption* and a *condensation* regime, respectively pertaining to the binding of water onto mineral surfaces and water film growth by water-water interactions. The Hygroscopic Growth Theory could also account for the particle size dependence on condensable water loadings under the premise that larger particles have a greater propensity of exhibiting of surface regions and interparticle spacings facilitating water condensation reactions. Our work should impact our ability to predict water film formation at mineral surfaces of contrasting particle sizes, and should thus contribute to our understanding of water adsorption and condensation reactions occuring in nature.

Mineral surfaces exposed to water vapour can stabilise thin water films (Fig. 1) of various degrees of organisation and thicknesses^1^,^2^,^3^, and their mechanisms of formation and growth are the object of an incessantly growing body of literature^4^. These films are of widespread occurence in nature, and play key roles in atmospheric, terrestrial and astronomical processes^5^,^6^,^7^,^8^. In the atmosphere, water films bound to mineral particle (dust) surfaces can impact cloud formation and activity^9^,^10^ as well as scattering and absorption of solar radiation^11^,^12^,^13^,^14^,^15^,^16^,^17^,^18^,^19^. In fact, while minerals represent only a fraction of all aerosols present in the atmosphere, they can be the prime nucleation sites upon which water and ice grow^19^. Water and ice films are also of strong relevance to aquatic and terrestrial environments, and even those of the Cryosphere where freeze-thaw cycles impact the fate of nutrients and contaminants, water cycling, as well as gaseous exchanges between terrestrial and atmospheric systems^20^. These interactions can even be of especial importance in the study of soil microorganisms inhabiting these films^21^. In outer-space, water and ice films are strongly relevant to the availability of water on planet Mars^5^,^19^, as well as to the catalytic transformations of gases (*e.g.*, CO_2_) in other planetary and cosmic bodies. Still, an ongoing challenge for all of these settings is to identify the mechanisms triggering water formation, growth and stability.

Given the importance of these mechanisms in nature, water binding is the object of extensive field and laborary investigations on environmentally and atmospherically relevant minerals (*e.g.* clays, quartz, feldspars, carbonates, Arizona Test Dust, volcanic ash)^4^,^7^,^18^,^22^,^23^,^24^,^25^,^26^,^27^,^28^. A recent review by Tang *et al*.^4^ provides a comprehensive view of this vast literature, and notably compares the ability of adsorption models at predicting water vapour binding in unsaturated and supersaturated atmospheres of water vapour. From a molecular view, we can regard water binding at hydrophilic surfaces of low-solubility minerals as a two-stage process. The first stage (*adsorption*) pertains to the attachment of water molecules to mineral surface functional groups via hydrogen bonding (Fig. 1), and should therefore strongly be controlled by mineral surface structural controls. Work with synthetic or purified natural samples is strongly beneficial in this regard, as we have demonstrated in the case of iron (oxyhydr)oxide minerals^2^,^29^,^30^, and revealed the strong impact of crystallographic orientation on the properties of thin nanometric water films. The second stage (*condensation*) is, in contrast, dominated by water-water interactions at mineral surfaces, and is a distinct process to homogenous water condensation. This can include growth and coalesence of water (nano)droplets and growth multiple layers of liquid water-like overlayers (Fig. 1). It also can occur at open surfaces or promoted within capillaries, in the interlayer of sheet minerals or interspaces of aggregated particles (Fig. 1). This stage should thus be largely independent of the identity of the mineral, but should obey the well-known Kelvin effect^31^ can be accounting the energetic contributions of water condensation at curved surfaces.

In an effort to attempt to generalise these concepts to a wide range of minerals of atmospheric and terrestrial relevance, we explored water vapour binding and condensation reactions on 21 samples of contrasting (i) mineral structure and (ii) composition, (iii) solubility, (iv) particle morphology/crystal habit (v) surface charge, and (vi) particle size/specific surface area (Supplementary Table 1). Minerals considered for this study (Fig. 2) were selected based on their importance in atmospheric and terrestrial systems^32^ and include (i) a suite of synthetic iron (oxyhydr)oxides of varied structure and particle habits and sizes, (ii) tectosilicates (quartz, microcline), (iii) a nesosilicate (olivine), (iv) expandable (montmorillonite) and non-expandable (kaolinite, illite) phyllosilicates, (v) calcium-magnesium carbonate, (vi) volcanic ash, and finally (vii) the widely-used Arizona Test Dust (ATD) for ice nucleation studies^18^,^23^,^27^,^28^,^33^.

In this work we explored the water-binding capabilities of these different minerals under ambient conditions using the Dynamic Vapour Sorption (DVS) technique. Quartz crystal microbalance (QCM) measurements of mineral particles exposed to water vapour revealed that particle size is the key parameter controlling water loadings deposited by condensation reactions. FTIR spectroscopy provided, at the same time, new insight into the hydrogen bonding environments adapted by thin water films in submicron- in relation to micron-sized minerals. These latter efforts notably build upon a recent study in our group focused on the properties of thin ice films formed in the same 21 mineral samples used of this study^34^. In this current study, we demonstrate the applicability of Hygroscopic Growth Theory (HGT)^35^ to account for the size dependence on water vapour binding in the 21 minerals under study, and discuss the implications and limitations of this and competing models in accurately accounting for molecular and thermodynamics aspects of the *adsorption* and *condensation* regimes.

## Results and Discussions

The water vapour pressure (p_w_) dependence on water loadings achieved by minerals at 25 °C was first monitored by QCM (Fig. 3). Our results readily revealed contrasting results between submicron and micron-sized particles. Submicron-sized particles clearly revealed *adsorption* and *condensation* regimes, expressed as *Type II* adsorption isotherms^36^. The *adsorption* regime can be seen mostly below ~12 Torr H_2_O where maximal loadings typically lie in the ~5–15 H_2_O/nm^2^ range. These loadings are comparable to crystallographic densities of reactive (hydr)oxo groups with which water vapour molecules form hydrogen bonds, and correspond to about one monolayer of water (*i.e.* 13–15 H_2_O/nm^2^ and ~0.28 nm thick, on a geometric basis). In contrast, the *condensation* regime is predominantly manifested at p_w_ where no more than 70 H_2_O/nm^2^, namely ~5 monolayers, are stabilised. The data do not reveal any clear contributions from differences in crystal habit, microporosity or surface (ζ) potential (Table S1). We also note that the largest water loadings achieved in submicron-sized particles occur in (1) ferrihydrite (a high specific surface area iron oxyhydroxide), (2) the bulk of akaganéite (β-FeOOH phase with the hollandite structure with nano-sized (4 × 4 Å) channels running along the length of particles), and in (3) the interlayer spacing of montmorillonites (expandable phyllosilicate minerals).

**Figure 1:**
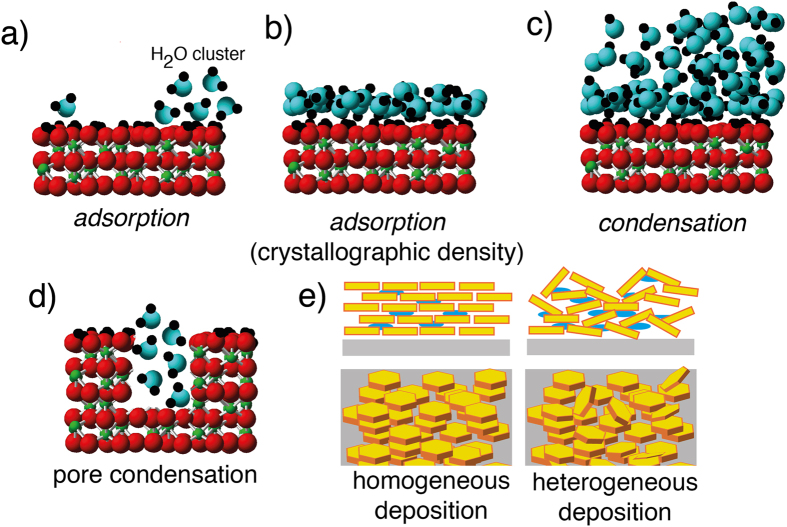
Schematic representation of water vapour binding at mineral surfaces.(**a**) The adsorption regime, also involving formation of water clusters. (**b**) Completion of the adsorption regime involving a monolayer. (**c**) The condensation regime dominated by water-water interactions. (**d**) Condensation of water in capillaries/pores of mineral surfaces. (**e**) Interparticle condensation of water in homogeneous (*e.g.* spray deposition) and heterogeneous (*e.g.* by rash deposition) of particles on a substrate.

**Figure 3:**
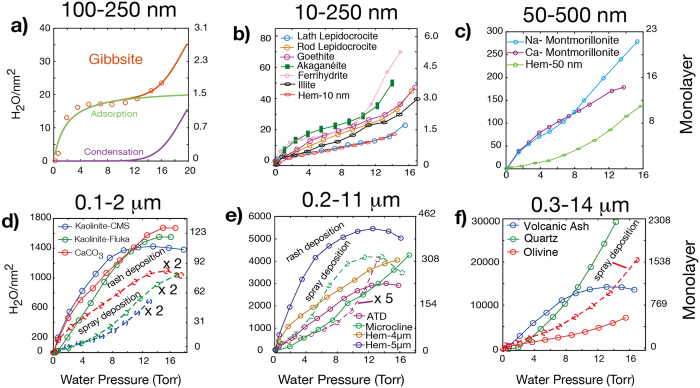
DVS (25 °C) results of mineral under study. (**a**) Gibbsite and Do-Do (Equation 4) modelling showing concurrent adsorption and condensation regimes. (**a**–**c**) Results for submicron-sized minerals, including expandable montmorillonite (**c**). (**d**–**f**) Results for micron-sized minerals.

In contrast, larger particles achieved more variable maximal loadings of the order of 1800–30000 H_2_O/nm^2^, namely about 120–2300 monolayers (Fig. 3d–f). As none of these loadings can be explained by filling of micropores — estimated by N_2_(g) adsorption/desorption isotherms (Supplementary Table 1) — these excess water molecules must reside at particle surfaces and even in the spacing between packed particles on the QCM electrodes which could catalyse condensation reactions. In fact, the method of deposition (*i.e.* heterogeneous coating by rash deposition *vs*. homogeneous coating by spray deposition, *cf.* Methods Section and Fig. 1) appear to affect water loadings as, for instance, seen in the case of kaolinite (Fig. 3d,e). Still, we must emphasise that loadings achieved by these contrasting deposition strategies in micron-sized particles remain at least one order of magnitude larger than those acquired on submicron-sized particles (Fig. 3a–c). We also note (i) the strongly contrasting loadings achieved by submicron-sized hematite (10 nm and 50 nm; Fig. 3b) compared to micron-sized hematite (4 μm and 5 μm; Fig. 3e), (ii) the congruent water loadings achieved by lepidocrocite (γ-FeOOH) particles of contrasting shapes (Fig. 3b), (iii) the contrasting loadings achieved in aluminosilicate minerals (montmorillonite, illite, kaolinite) shaped as platelets and with strongly-expressed basal faces, and (iv) a possibly larger water uptake in the K-feldspar-bearing kaolinite (Fluka) than in the purer kaolinite (CMS-KGa-1b) preparations. From these results we can thus largely discard the impact of (i) mineral structure, (ii) composition, (iii) particle morphology/crystal habit and (iv) surface potential (namely ζ-Potential as could be acquired in aqueous media) as major factors driving the water loadings measured by DVS (Supplementary Table 1).

**Figure 4:**
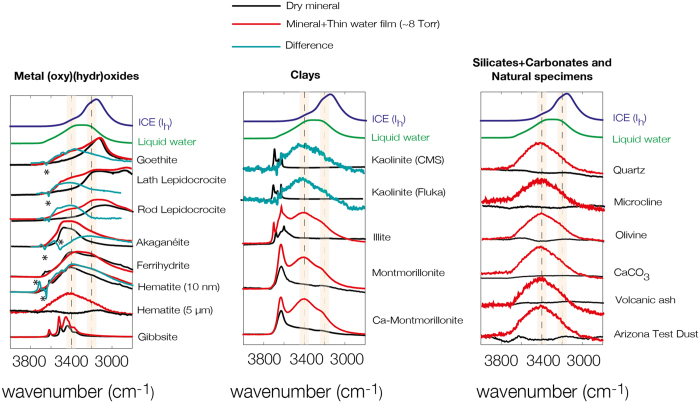
Representative FTIR spectra of thin water films at mineral surfaces at 25 °C. See example of water vapour binding experiment for the case of illite in Fig. S1. The symbols “*” in goethite, lath lepidocrocite, rod lepidocrocite and akaganéite denote losses in surface^2^,^29^,^30^ (and bulk in akaganéite, *cf.* Song and Boily^57^) OH groups caused by water binding.

FTIR spectroscopy revealed additional insight into the nature of the mineral-bound water films under study (Fig. 4). In all cases did the micrometer-sized particles exhibit highly comparable distributions of O-H stretching frequencies, namely with an intense band at ~3400 cm^−1^ and a relatively attenuated band at ~3200 cm^−1^. These spectra can be used to suggest that mineral-bound water molecules form a smaller number and weaker hydrogen bonds than in liquid water. This result can be explained by the relatively thin (*e.g.* ~1.4 nm for 5 and ~277 nm for 1000 evenly spread monolayers) water films formed at these surfaces, whose structure are less amenable to water-water interactions than in liquid water. In particular thin water (1–3 monolayer) films embedded in the intralayers of montmorillonite exhibit these features. At the same time, the nearly symmetric bending region at ~1610–1640 cm^−1^ (not shown) is generally comparable to that of liquid water. Thus, the general spectral features of water films adopted by the large particles are comparable with one another, yet not entirely comparable with liquid water or hexagonal ice (I_h_)^37^. The smaller submicron-sized particles exhibited more unique spectral features tailored by the intricate hydrogen bonding interactions between mineral surface (hydr)oxo groups and waters (Fig. 4), as well as discrete bands resulting from surface hydroxo groups. While more detailed discussions on these specific features are best achieved in communications dedicated to these issues, such as in our previous work in iron (oxyhydr)oxides^2^,^29^,^30^, we note that the weaker intensities at the lower O-H stretching frequencies (*e.g.* ~3200 cm^−1^) denote smaller extents of water-water interactions in these thinner films.

**Figure 5:**
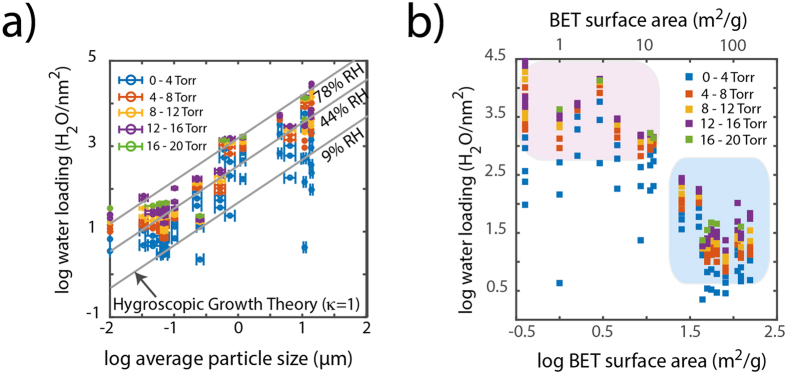
Water loading dependence on (**a**) particle size and (**b**) specific surface area of the 21 minerals under study. The Hygroscopic Growth Theory using κ = 1 provides a general description of the condensable water loadings achieved at mineral particles (Equations 1, 2, 3) log_10_.

The strongest relationship that could be umambiguously identified between the physicochemical properties of the minerals (Supplementary Table 1), and DVS data (Fig. 3) pertains to particle size (Fig. 5). This relationship is shown for the larger fractions of particles that could be imaged by electron microscopy, and is exponential-like over 3–4 orders of magnitude of values. A log-log_10_ plot of these values (Fig. 5a) underscores the clear impact of particle size to water loadings especially at pressures exceeding 4 Torr H_2_O (~17% RH, relative humidity). This finding thus falls in line with current pratices in the atmospheric modelling community^38^ considering particle size as a parameter for modelling cloud droplet size, aerosol growth and transformation processes^38^.

A formulation of the Hygroscopic Growth Theory (HGT)^35^ previously developed to undersaturated conditions can readily account for this relation (Fig. 5a). HGT describes water loadings (Θ; *e.g.* H_2_O/nm^2^) in terms of differences in the diameter of wet (D) in relation to dry (D_d_) particles^39^:


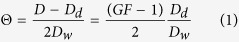


through a so-called growth factor defined as GF = D/D_d_, and assuming a given diameter D_w_ for a single water molecule (*e.g.* 0.277 nm for a monolayer density of 13 H_2_O/nm^2^). At the base of the calculation of GF is the equation predicting the impact that the particle plays on the activity of water (a_w_) through the unitless hygroscopic parameter κ:


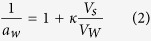


expressed by the ratios of the particulate matter (V_s_) and water (V_w_) volumes. The introduction of particulate matter to a fixed volume of water should thus decrease a_w_ because mineral-water interactions perturb the structure and hydrogen bond populations and dynamics of interfacially-bound water molecules, and even possibly the energetics (*cf.* surface tension) of the air/water interface^40^. GF in HGT developed for unsaturated conditions, and for a given geometric relationship between D and V, is obtained through:


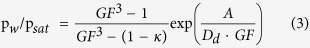


where p_sat_ is the saturation pressure at a given temperature, and p_w_/p_sat_ = a_w_ is relative humidity expressed as a fraction. This function also accounts for the Kelvin effect on the energetics of water condensation at curved surfaces (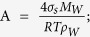 where σ_s_ is surface tension (0.072 J ·m^−2^ at 25 °C), M_W_ is molecular weight and ρ_w_ the density water), which is namely effective in particles below ~100 nm in diameter. Because many of the minerals under study are of only negligible to low solubility, only a fraction of the particulate matter actually interacts with mineral surfaces, thus potentially raising uncertainties as to how the V_s_/V_w_ ratio in Eq. 2 relates to the ability of minerals in altering a_w_, as will be discussed in the latter part of this article. Still, the HGT provides a very reasonable description of the QCM-derived DVS adsorption data (Fig. 5a) with a value of κ≈1 over the different p_w_ values explored in this work. It could, as such, be a recommended value for modelling water condensation at clean mineral surfaces of varied particle size, structure, composition and even surface charge. Certainly, this approach makes the most sense for field-based applications (*e.g.* atmospheric aerosol chemistry, vadose zone biogeochemistry) involving heterogeneous mixtures of minerals.

**Figure 6:**
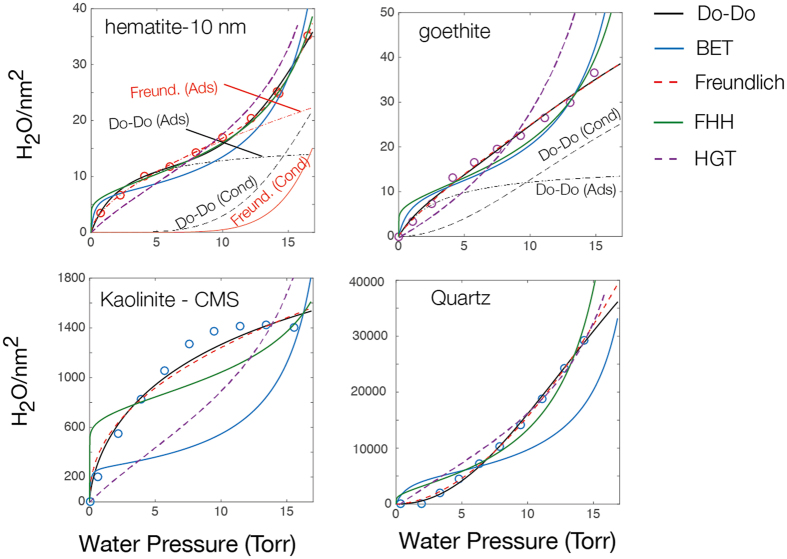
Best-fits of five dominant models describing water binding on solid surfaces. Fits were obtained by non-linear least square optimisation of model parameters. Equations for the Do-Do (Equation 4), BET, Freundlich, FHH and HGT (Equations 1, 2, 3) are briefly discussed in the Supplementary Section.

Although the HGT provides a means at approximately predicting the *condensation* regime in the larger-sized particles, it does not accurately predict Type II^36^ adsorption isotherms of the smaller particles as a function of water vapour pressure, where both *adsorption* and *condensation* regimes are clearly expressed (*e.g.* Fig. 6). One possibility that arose during our modelling attempts is to make use of exceeding large κ (*e.g.* 10–1000) and to scale the resulting Θ values to fit the data. Still, concerns may readily be raised as to how this paramerisation relates to the physicochemical reality of water condensation. Comparison with more classical formulations (*cf.* Brunauer-Emmett-Teller^41^, Freundlich^42^, Frenkel-Halsey-Hill^43^,^44^,^45^), and that were notably recently reviewed by Tang *et al*.^4^ (*cf.* Supplementary information for a synopsis), reveal the strong predictive capability of 2-term Freundlich^42^ model in predicting both the *adsorption* and *condensation* regimes. In addition to this, we value the predictive capability of the Do-Do^46^ model in describing these regimes in a framework that relates to plausible molecular-scale processes. Although this latter model was originally intended to predict water vapour uptake in microporous carbon, it can be readily adapted to the case of binding onto mineral surfaces (*cf.* Fig. 3a for gibbsite) in the following manner. In the Do-Do^46^ formulation:


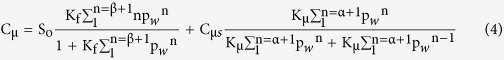


the *adsorption* (left-hand) and *condensation* (right-hand) terms are explicitely taken into account with parameters including water-binding sites densities (S_o_, C_μs_), association constant (K_f_, K_μ_) and hydration numbers (β, α). In this approach the *adsorption* regime is predicted with S_o_ values contrained to crystallographic densities of surface (hydr)oxo groups (*e.g.* 10–15 nm^−2^) and with a hydration number fixed to β≈2 (or any other estimate) to denote the number of hydrogen bonds involved with first layer water molecules, as often suggested by molecular modelling^40^. The *condensation* regime occurs at a p_w_ where water nanoclusters of a given population (*e.g.* α≈6)^46^ is achieved.

**Figure 7:**
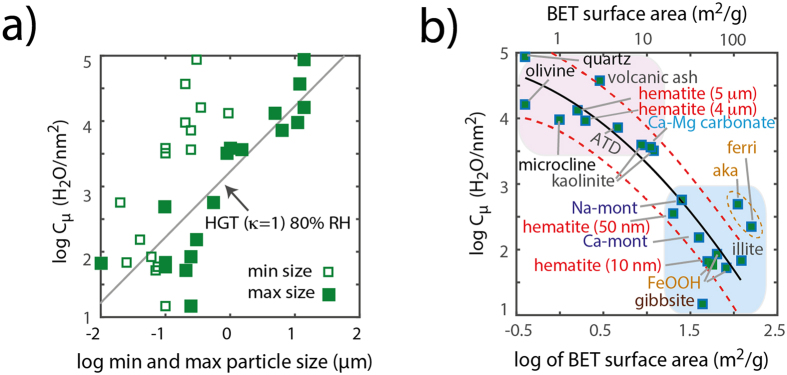
Condensation saturation densities obtained by fitting of DVS data (Fig. 3) with the Do-Do model (Equation 4) as a function of lower and upper ranges of particle size (**a**) and specific surface area (**b**). These latter data can be modeled using the function log(H_2_O/nm^2^) = −0.29 (log(s_s_))^2^ – 0.76 log(s_s_) + 4.36, where s_s_ is specific surface area in m^2^/g. The dashed lines show the model predictions within ~1σ. ‘aka’ is the abbreviation for ‘akaganéite’ and ‘ferri’ for ‘ferrihydrite’. These two FeOOH-like minerals have slightly larger water loadings due to the incorporation of water in the bulk structure of akaganéite^57^ and possible condensation in aggregated nano-sized ferrihydrite particles log_10_.

C_μs_ values derived from this model provide an effective means at summarising the water condensation loadings achieved in the 21 mineral samples under study (Fig. 7). We first note that these values are strongly congruent with particle size (Fig. 7). They can also be roughly predicted, very much like the raw data of Fig. 5, by HGT with κ = 1 when expressed as a function of the larger-sized particles (Fig. 7a) but much not for the smaller size fractions (Supplementary Table 1). An even stronger correlation can be made by expressing these values as a function of specific surface area (s_s_), and notably where a steep dependence with particles below ~10 m^2^/g (Fig. 7b) reinforces further the idea that condensation reactions are disfavored in the submicron-sized particles. This even raises even further the importance of recognising that the smallest dimensions of anisotropically-shaped particles (*e.g.* acicular FeOOH minerals of 5–20 nm in width and a few hundred nm in length) could be the parameter needed to evaluate the extent to which the Kelvin^31^ effect applies to these surfaces.

These observations bring us back to how GF of Eq. 3 is related to the physicochemical reality of the mineral/water interface. As most of the minerals under study are of only negligible to low solubility, release of soluble ions that could potentially decrease the enery barrier for water condensation and/or alteration of surface tension are, in our view, largely limited. In the great majority of the minerals under study only a fraction of the particulate matter actually interacts with mineral surfaces. It is consequently only this fraction, and not the embedded atoms composing the bulk, that influences interfacial water structure, hydrogen bonding populations and dynamics that are collectively affecting water activity. The D_d_ term could consequently be a proxy comensurating with the propensity of physicochemical features promoting water condensation in micron-sized particles. These features would include nano- to micron-scale surface roughness (*e.g.* steps, hillocks, crevasses, pores)^47^ and interparticle spacing (*e.g.* in aggregated particles, kaolinite booklets, *etc*.) in aggregated materials, but that have yet to be unambiguously measured and/or observed (e.g. via microscopy). An empirical approach based on the experimental data of this study involves predicting condensable densities of water through:


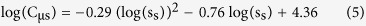


and with values of s_s_ (m^2^/g) related to particle diameter (D_d_ in m), through the data shown in Supplementary Figure 1, with:


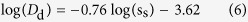


GF can then be generated from p_w_/p_sat_ using D_d_ in Eq. 3. Still, a future reformulation^48^,^49^,^50^,^51^ of the HGT relating the occurence of sites or regions promoting water condensation to particle size could be a viable strategy for bridging this theory to (molecular-based) adsorption isotherm models. This would represent a much needed step for securing our ability at predicting water condensation at mineral surfaces and their aggregates.

## Conclusions

This study confirms further the notion that water loadings achieved by vapour condensation is strongly controlled by particle size. Micron-sized particles with specific surface area less than ~10 m^2^/g promote water condensation while submicron-sized particles stabilise water films formed by adsorption. Submicron-sized particles cannot promote condensation reactions due the relative paucity of surface regions of interparticle spaces promoting condensation reactions, and due to the otherwise expected Kelvin effect. Water adsorption isotherms in all 21 mineral under study are best described using (1–2 term) Freundlich and Do-Do models, and the particle size dependence by the HGT model. These findings should consequently help constrain further efforts in advancing knowledge water vapour condensation reactions at surfaces, as well as in evaluating the impact that co-existing solids (*e.g.* sea sprays), overcoatings (*e.g.* salts) or even reactive gases (*e.g.* NO_x_, SO_x_, CO_2_) play in this regard.

In particular, we anticipate that these results should be strongly relevant to advancing our knowledge of atmospheric cloud formation processes via condensation reactions of cloud droplets on aerosol particles even under supersaturated conditions^4^,^38^,^52^,^53^. Activation of these so-called cloud condensation nuclei are the object of intense field and laboratory studies, and could be strongly related to particle size, as notably emphasised in a number of studies^4^,^38^,^53^,^54^, and surface defects (e.g pores, kinks, roughness)^7^,^54^, but much less so to the composition of insoluble minerals. This being said, we can anticipate strong departures from this statement for mixed systems containing dissolvable salts (*e.g.* metal oxide-sea spray salts), or under conditions of photo-, proton- and/or ligand-promoted mineral dissolution^55^. Additionally, the impact of particle aggregation on water vapour condensation, such as in the lines of our DVS data with particle deposited by spray *vs.* rash deposition (Fig. 1), warrants further investigations. These possibilities should consequently be more explicitely addressed in future studies with the goal of refining the impact of particle size and chemical composition in the accurate prediction of water adsorption and condensation isotherms such as those presented in this study.

## Methods

### Minerals and Characterization

All metal (oxyhydr)oxides were synthetised in our laboratory using well-established methods for goethite^56^ (α-FeOOH), rod- and lath-shaped lepidocrocite^56^ (γ-FeOOH), akaganéite (β-FeOOH)^57^, ferrihydrite^56^ (*e.g.* Fe_8.2_O_8.5_(OH)_7.4_ + 3 H_2_O), gibbsite^58^ (γ-Al(OH_3_)), as well as nano-sized^56^ and micron-sized^59^ hematite (α-Fe_2_O_3_). Quartz (α-SiO_2_), olivine ((Fe,Mg)_2_SiO_4_) were taken from the mineral collection of Umeå University and ground to a fine powder with an agate mortar and pestle. Powdered forms of microcline (KAlSi_3_O_8_) were obtained from Technical University Darmstadt, and kaolinite (Al_2_Si_2_O_5_(OH)_4_) were obtained from Fluka (Sigma Aldrich) and from the Clay Mineral Society (KGa-1b). Illite-rich (K,H_3_O)(Al,Mg,Fe)_2_(Si,Al)_4_O_10_[(OH)_2_,(H_2_O)] powder was obtained from Arginotec (B + M Nottenkämper), while Na-montmorillonite was obtained (Ca_0.52_Na_0.14_K_0.01_)(Al_3.23_Fe^3+^_0.42_Mn_0.01_Mg_0.56_)(Si_7.89_Al_0.11_)O_10_(OH)_2_; (SWy-2) from the Clay Mineral Repository, an a portion was Ca-exchanged. Calcium carbonate was obtained from KEBO Lab AB, Arizona Test Dust (ATD) from (Ultrafine Test Dust, Powder Technology Inc.), and volcanic ash from Eyjafjallajökull (Iceland).

Salient physical and chemical properties of these minerals are presented in Supplementary Table 1. Phases made in the laboratory were confirmed by our own powder X-ray diffraction (Bruker d8 Advance working in θ−θ mode with Cu Kα radiation) measurements. Those that were acquired were already characterised for phase purity and crystallinity, when applicable, by our providers. All mineral surfaces were tested for surface elemental composition using X-ray photoelectron spectroscopy (Kratos Axis Ultra DLD electron spectrometer). The results of these XPS analyses (Supplementary Table 1) notably show that surfaces are strongly representative of their bulk composition and contain little organic impurities (not shown).

Particle sizes were estimated by imaging using Scanning Electron Microscopy (SEM; Zeiss Merlin, GmbH) or Transmission Electron Microscopy (TEM; JE-1230 (JEOL)) (Fig. 1). Up to 5 different particles in each of 3 to 7 different images, and at various magnifications where needed, were investigated to collect information on the distribution of particles sizes, the results of which are presented in Supplementary Table 1. Specific B.E.T. specific surface area and B.J.H. micropore volumes were obtained by 90-point N_2_(g) gas adsorption/desorption isotherms (Micromiretics) at LN2. Micropore volumes were used to estimate maximal levels of pore water. Supplementary Figure 1 also shows a correlation between B.E.T. specific surface area and particle size estimated by imaging and predicted via Eq. 6. Finally, surface (ζ) potentials of mineral particles were determined at their natural pH of suspension at 25 °C by electrophoresis (Zetasizer, Malvern).

### Dynamic Vapour Sorption

Water vapour uptake by minerals was measured by Quartz Crystal Microbalance (QCM; eQCM 10M, Gamry Instruments Inc.), using with the DVS method at 25 °C. The serial resonance frequency (*f*_s_) of a 10 MHz gold-coated quartz resonator was first determined by measurements under a constant total flow rate of 200 standard cubic centimeters per minute (sccm) of dry N_2_(g). A mass flow controller (MKS, 179A) was used to control this gas flow. The crystal was then coated by pipetting or spraying a dilute aqueous suspension of minerals over the gold area, then drying under a stream of 200 sccm dry N_2_(g). Montmorillonite samples were dried overnight to remove the interlayer waters. The crystal was then emplaced back into the measurement cell and equilibrated under 200 sccm N_2_(g) for at least 1 h, after which time *f*_*s*_ was determined to obtain the dry, time-independent, weight of the mineral sample. The parallel resonance frequency (*f*_p_) was tracked to monitor the viscosity of the mineral films on the quartz resonator. Correlated *f*_p_ and *f*_s_ values resulted from thin homogeneous films (Fig. 1e, left) and were observed for FeOOH minerals, gibbsite and illite deposited by pipetting, as well as micron-sized minerals deposited by spraying. Uncorrelated *f*_p_ and *f*_s_ values resulted from thicker heterogeneous films (Fig. 1e, right) produced by pipetting of micron-sized particles. This was confirmed further by optical microscopy.

Water vapour adsorption isotherms were then collected by monitoring the frequency of the mineral-coated QCM crystal exposed to a steady stream of 200 sccm N_2_(g) + H_2_O(g) in the 0–18 Torr H_2_O range. Water partial pressure was generated by blending streams of dry and water-saturated N_2_(g) using mass-flow controllers, and monitored with a calibrated Non Dispersible Infrared device (LI-7000, Licor Inc.). This device was also used to ensure that the gases were free of CO_2_(g). The samples were equilibrated to a fixed partial pressure of water for a 20 to 30 min period to ensure that *f*_*s*_ values were time-independent. The Sauerbrey equation was used to convert *f*_s_ values of the QCM crystal to masses of samples (3.5–41 μg) under N_2_(g) conditions, and water loadings. Additionally, thin film rigidity was tracked with the parallel resonator frequency (*f*_*p*_) to ensure that the viscocity on water vapour sorbed film is sufficiently large for the measurements to be feasible.

FTIR spectra of water vapour sorption at mineral surfaces were collected using an Attenuated Total Reflectance (ATR) accessory (Golden Gate, Specac). Aqueous suspensions of the minerals were centrifuged at their natural pH and then dried directly on the single-bound diamond ATR cell under a stream of N_2_(g). Samples were then covered with a flow-through cell and exposed to partial pressures of water vapour using the same experimental protocol as in the QCM experiments. All spectra were collected *in-situ* using a Bruker Vertex 70/V FTIR spectrometer, equipped with a DLaTGS detector, in a room kept at 25 °C. The spectra were collected in the 600–4000 cm^−1^ spectral range at 4 cm^−1^ resolution, and with a forward/reverse scanning rate of 10 Hz. Background spectra were collected with the help of gas flow under 200 sccm N_2_(g). We used the Blackman–Harris three-term apodisation function with 16 cm^−1^ phase resolution and the Mertz phase correction algorithm. Each spectrum was obtained from 100 scans, each collected over a 89 sec period. A chemometric analysis of the resulting spectra (*cf.* Fig. S2 for an example with illite) involved the multivariate curve resolution method^60^ extracting representative spectral components of mineral-bound water films. This method was especially employed for submicron-sized particles, and the spectra shown in Fig. 4 are representative of films at ~8 Torr H_2_O(g).

## Additional Information

**How to cite this article**: Yeşilbaş, M. and Boily, J.-F. Particle Size Controls on Water Adsorption and Condensation Regimes at Mineral Surfaces. *Sci. Rep.* **6**, 32136; doi: 10.1038/srep32136 (2016).

## Supplementary Material

Supplementary Information

## Figures and Tables

**Figure 2:**
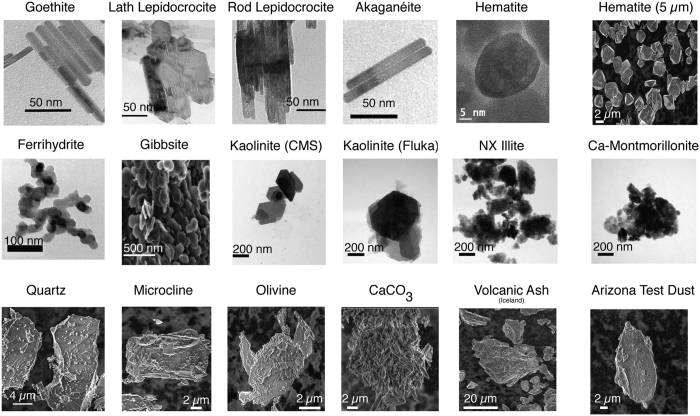
SEM and TEM images of minerals under this study.
