# Supplementary material for: Particle Size Controls on Water Adsorption and Condensation Regimes at Mineral Surfaces
Source: Sci Rep. 2016 Aug 26;6:32136. doi: 10.1038/srep32136 (PMC5000481; doi:10.1038/srep32136)
Supplement: Supplementary Information [file srep32136-s1.pdf]

# SUPPLEMENTARY MATERIAL

# Particle Size Controls on Water Adsorption and Condensation Regimes at Mineral Surfaces

Merve Yeşilbaş, Jean-François Boily*

Department of Chemistry, Umeå University, SE-901 87 Umeå, Sweden

*tel. +46 73 833 2678; email jean-francois.boily@chem.umu.se

## 1. Modeling of Dynamic Vapor Sorption Data

The following sections detail the salient equations used to predict water vapor binding as a function of $p_w/p_{sat}$ where $p_w$ is the partial pressure of water vapour and $p_{sat}$ is the pressure at saturation (23.76 Torr at 25°C)

*1.1 Bruauner-Emmet-Teller (BET)*

The well-known BET[1] equation typically applied for determination of specific surface area using $N_2(g)$ is written as:

$$C_\mu = S_o \frac{p_w/p_{sat} \cdot c}{(1-p_w/p_{sat})(1-p_w/p_{sat}+c \cdot p_w/p_{sat})} \quad (1)$$

where $S_o$ is the water-binding site density, and $c = e^{(\Delta_{des}H^0 - \Delta_{vap}H^0)/RT}$, namely where $\Delta_{des}H^0$ and $\Delta_{vap}H^0$ are the standard enthalpy of water desorption and evaporation, respectively.

*1.2 Freundlich*

The single-site Freundlich[2] equation

$$C_\mu = S_o \sqrt[K]{p_w/p_{sat}} \quad (2)$$

where K is the adsorption constant, and where $S_o$ is a site density. It can an be readily adapted to the multisite case through:

$$C_{\mu,tot} = \sum_i C_{\mu,i} \quad (3)$$

and effectively mimic the Do-Do[3] equation (Section 1.4) by providing a means to predict an *adsorption* regime at low $p_w/p_{sat}$ and a *condensation* regime at high $p_w/p_{sat}$.

*1.3 Frenkel-Halsey-Hill*

This formulation is expressed as[4-8]:

$$C_\mu = S_o \sqrt[K2]{\frac{K1}{-\ln(p_w/p_{sat})}} \quad (4)$$

where $K_l$ pertains to interactions between the mineral surface and the first water layer, while $K_2$ with longer range water molecules on thicker water layers.

*1.4 Do and Do*

This model was originally developed to predict water vapor *adsorption* and *condensation* in carbon-based materials through the following equation[3]:

$$C_\mu = S_o \frac{K_f \sum_1^{n=\beta+1} n p_w{}^n}{1+K_f \sum_1^{n=\beta+1} p_w{}^n} + C_{\mu s} \frac{K_\mu \sum_1^{n=\alpha+1} p_w{}^n}{K_\mu \sum_1^{n=\alpha+1} p_w{}^n + K_\mu \sum_1^{n=\alpha+1} p_w{}^{n-1}} \quad (5)$$

The left-hand term pertains to the *adsorption* and the right-hand term to the *condensation* regime which, we argue, could be translated to the case of water vapor adsorption and condensation at mineral surfaces. Parameters for each regime include water-binding sites densities ($S_o$, $C_{\mu s}$) association constant ($K_f$, $K_\mu$) but also hydration numbers ($\beta$,$\alpha$). The latter numbers are fixed to $\beta$=2 for the *adsorption* regime, to denote that a singly (hydr)oxo group can be involved in 2 (donating and/or accepting) hydrogen bonds, and $\alpha$=6 for the *condensation* regime to denote that the nominal population of a water nanocluster needed for condensation at the mineral surface. These numbers may optionally be co-optimized to predict adsorption data, yet must be confined to physically realistic values.

**Supplementary Table 1.** Salient chemical and physical properties of minerals under study.

| Mineral name | Atomic Ratio by XPS[a] | Particle Size[b] | Average Particle Size[b] | B.E.T Surface Area ($m^2/g$) [c] | B.J.H Micropore volume ($cm^3/g$)[d] | Maximal pore water (mg $H_2O/m^2$ mineral)[e] | Maximal pore water ($H_2O$ sites/$nm^2$ mineral)[e] | ζ-potential (mV) [f] |
|---|---|---|---|---|---|---|---|---|
| Goethite | **Fe:O:OH =** 1.35:1.0:1.43 | 75-100 nm | 75 ± 9.4 nm | 55.6 | 0.085 | 0.085 | 158 | 47.5 |
| Lath-Lepidocrocite | **Fe:O:OH =** 1.4:1.0:1.2 | 70-210 nm | 70 ± 9 nm | 81.3 | 0.124 | 0.085 | 230.4 | 10 |
| Rod-Lepidocrocite | **Fe:O:OH =** 1.3:1.0:1.2 | 60-250 nm | 60 ± 7.1 nm | 64.4 | 0.204* | 0.176* | 379* | 7 |
| Akaganéite | **Fe:O:OH:Cl** = 1.0:0.82:1.22:0.17 | 100 nm | 102 ± 11 nm | 111.2 | 0.22 | 0.110 | 408.7 | 43 |
| Ferrihydrite | **Fe:O:OH =** 1.5:1.3:1.0 | ~ 25-50 nm | 34 ± 5.4 nm | 155 | 0.051* | 0.018 | 95 | 10.5 |
| Hematite (10 nm) | **Fe:O:OH =** 4.1:4.5:1.0 | 10 nm | 10 ± 0.01 nm | 50 | 0.182* | 0.202 | 338.2 | 32.1 |
| Hematite (50 nm) | **Fe:O:OH** = 2.8:3.88:1.0 | 50 nm | 50 ± 6 nm | 20.4 | 0.093 | 0.253 | 172.8 | 38.4 |
| Hematite (4μm) | **Fe:O:OH** = 1.5:1.9:1.0 | 1-4 μm | 3.66 ± 0.82 μm | 2 | 0.064 | 1.77 | 118.9 | 10.1 |
| Hematite (5μm) | **Fe:O:OH** = 1.7:2.0:1.1 | 0.95-5 μm | 4.5 ± 0.6 μm | 1.6 | 0.048 | 1.66 | 89.2 | 37.4 |
| Gibbsite | **Al:OH =** 1.0:3.0 | 100-290 nm | 255 ± 35 nm | 44 | 0.26 | 0.328 | 483.1 | 40.3 |
| Kaolinite (CMS) | **K:Al:Si:O** = 0.0:2.0:2.0:8.2 | 100-900 nm | 600 ± 110 nm | 12 | 0.126 | 0.583 | 234.1 | - 11 |
| Kaolinite (Fluka) | **K:Al:Si:O** = 0.2:2.0:2.6:9.3 | 0.1-1 μm | 745 ± 15 nm | 8.6 | 0.121 | 0.782 | 225 | - 22.4 |
| Illite | **K:Al:Si:O** = 0.3:1.0:2.5:7.6 | 25-100 nm | 50 ± 15 nm | 121.7 | 0.228 | 0.104 | 424 | - 11.2 |
| Na-Montmorillonite | **Na:Fe:Mg:Al:Si:O** = 0.1:0.1:0.1:1.0:2.3:7.8 | 20-550 nm | 520 ± 110 nm | 25.3 | 0.138 | 0.303 | 256.4 | - 26.3 |
| Ca-Montmorillonite | **Ca:Fe:Mg:Al:Si:O** = 0.1:0.1:0.1:1.0:2.3:8.2 | 40-300 nm | 230 ± 40 nm | 39.8 | 0.191 | 0.266 | 355 | - 18.3 |
| Quartz | **Si:O** = 1.0: 2.0 | 0.3-14 μm | 14 ± 0.7 μm | 0.4 | 0.024* | 3.33* | 45* | - 32.7 |
| Microcline | **Na:K:Al:Si:O** =0.2:0.8:1.0:2.8:7.2 | 0.2-11 μm | 11 ± 0.5 μm | 1 | 0.041 | 2.27 | 76.2 | - 45.2 |
| Olivine | **Fe:Mg:Si:O** = 0.1:0.95:1.0:4.0 | 0.35-13.7 μm | 13.7 ± 0.7 μm | 0.4 | 0.005 | 0.69 | 9.3 | - 20.9 |

| | | | | | | | | |
|---|---|---|---|---|---|---|---|---|
| Calcium carbonate | **Mg:Ca:C:O** = 0.3 : 0.7 : 1.1 : 3.6 | 0.25-1.5 μm | 1.2 ± 0.12 μm | 11 | 0.057 | 0.28 | 106 | 12.7 |
| Volcanic ash | **Na:K:Ca:Mg:Fe:Al:Si:O** = 0.4:0.1:0.3:0.3:1.0:1.0:3.4:12.5 | 0.2-12 μm | 11 ± 1.6 μm | 2.9 | 0.011 | 0.21 | 20.4 | - 24.3 |
| Arizona Test Dust (ATD) | **Na:K:Mg:Fe:Al:Si:O** = 0.2:0.3:0.1:0.1:1.0:7.1:19.3 | 0.25-6.4 μm | 6.4 ± 1.5 μm | 4.6 | 0.06 | 0.725 | 111.5 | -24.1 |

a. *In vacuo* XPS measurements:
b. Size range obtained by SEM or TEM imaging;
c. From B.E.T. analysis of 90-point $N_2$(g) adsorption/desorption isotherms at $LN_2$;
d. From B.J.H. analysis of 90-point $N_2$(g) adsorption/desorption isotherms at $LN_2$;
(* Single point adsorption total pore volume of pores)
e. Derived from B.J.H pore volume.
f. Obtained from electrophoretic mobility of 2g/L suspensions of particles in distilled deionized water at 298 K.

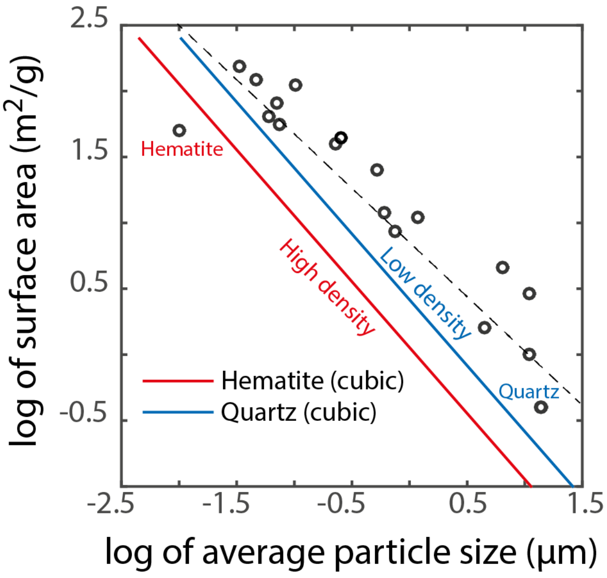


**Supplementary Figure 1.** Relationship between $N_2$-BET specific surface area ($s_s$ in $m^2/g$) and particle size ($D_d$ in nm) estimated by scanning and transmission electron imaging. Both parameters can be related with the empirical function (dashed line) $\log(D_d) = -0.76 \log(s_s) - 3.62$ where $s_s$ is in $m^2/g$ and $D_d$ is in m. Theoretical predictions assuming cubic-shaped particles of quartz (density of 2.6 $g/cm^3$) and hematite (density of 5.3 $g/cm^3$) underestimate the experimentally measured $N_2$-BET values. Discrepancies between theoretical and experimental values are caused by a bias in the sampling of the larger-sized particles, as well as micropore surface area.

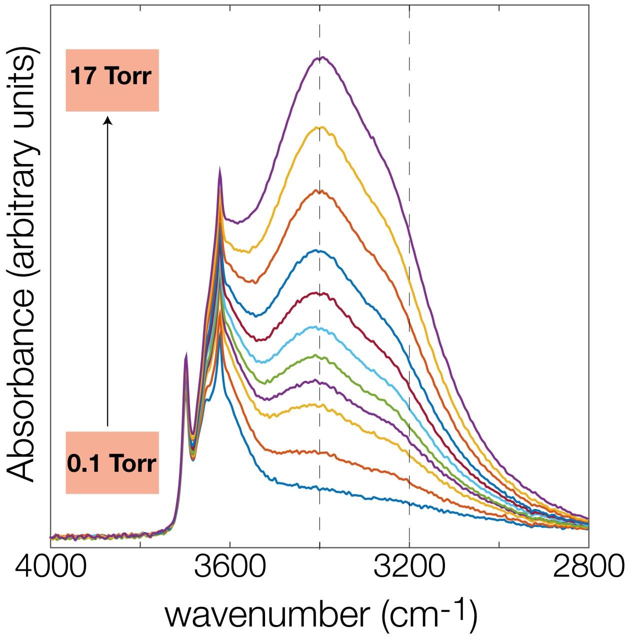


**Supplementary Figure 2.** Example of FTIR spectra of illite in the O-H stretching region during water vapor adsorption at 25 °C.

## References


1 Brunauer, S., Emmet, P. H. & Teller, A. Adsorption of gases in multimolecular layers. *J. Am. Chem. Soc.* **60**, 309-319 (1938).

2 HMF, F. Ûber die Adsorption in Lösnungen. *Z. Phys. Chem.* **57**, 385-470 (1906).

3 Do, D. D. & Do, H. D. A model for water adsorption in activated carbon. *Carbon* **38**, 767-773, doi:10.1016/s0008-6223(99)00159-1 (2000).

4 J., F. *Kinetic Theory of Liquids*. (Oxford University Press, 1946).

5 Halsey, G. Physical Adsorption on Non-Uniform Surfaces. *J Chem Phys* **16**, 931-937, doi:doi:http://dx.doi.org/10.1063/1.1746689 (1948).

6 Hill, T. L. Statistical Mechanics of Multimolecular Adsorption II. Localized and Mobile Adsorption and Absorption. *J Chem Phys* **14**, 441-453, doi:doi:http://dx.doi.org/10.1063/1.1724166 (1946).

7 Hill, T. L. Statistical Mechanics of Multimolecular Adsorption. III. Introductory Treatment of Horizontal Interactions. Capillary Condensation and Hysteresis1 a. *J Chem Phys* **15**, 767-777, doi:doi:http://dx.doi.org/10.1063/1.1746330 (1947).

8 Hill, T. L. Extension of Fowler's Treatment of Surface Tension to Physical Adsorption. *J Chem Phys* **17**, 668-669, doi:doi:http://dx.doi.org/10.1063/1.1747364 (1949).

9 Petters, M. D. & Kreidenweis, S. M. A single parameter representation of hygroscopic growth and cloud condensation nucleus activity. *Atmos. Chem. Phys.* **7**, 1961-1971, doi:10.5194/acp-7-1961-2007 (2007).

10 Sorjamaa, R. & Laaksonen, A. The effect of H2O adsorption on cloud drop activation of insoluble particles: a theoretical framework. *Atmos Chem Phys* **7**, 6175-6180 (2007).

11 Boily, J. F. Water structure and hydrogen bonding at goethite/water interfaces: implications for proton affinities. *J. Phys. Chem. C* **116**, 4714-4724, doi:Doi 10.1021/Jp2110456 (2012).
